# PhTx3-4, a Spider Toxin Calcium Channel Blocker, Reduces NMDA-Induced Injury of the Retina

**DOI:** 10.3390/toxins8030070

**Published:** 2016-03-11

**Authors:** Nancy Scardua Binda, Charles Porto Petruceli Carayon, Rafael Mourão Agostini, Ana Cristina do Nascimento Pinheiro, Marta Nascimento Cordeiro, Marco Aurélio Romano Silva, Juliana Figueira Silva, Elizete Maria Rita Pereira, Claudio Antonio da Silva Junior, Célio José de Castro Junior, Andre Luiz Sena Guimarães, Marcus Vinicius Gomez

**Affiliations:** 1 Institute of Education and Research Santa Casa Belo Horizonte-Laboratory of Toxins, Rua Domingos Vieira 590, Belo Horizonte, Minas Gerais 30150-240, Brazil; nancysbinda@yahoo.com.br (N.S.B.); charlespetruceli@hotmail.com (C.P.P.C.); rafaelagostini@uol.com.br (R.M.A.); anapinheirofar@gmail.com (A.C.N.P.); jufigueira2003@yahoo.com.br (J.F.S.); elizetemrpereira@yahoo.com.br (E.M.R.P.); claudiojunior.biologia@gmail.com (C.A.S.J.); celiojcjunior@gmail.com (C.J.C.J.); 2 Ezequiel Dias Foundation (FUNED), Laboratory of Biochemistry, Rua Conde Pereira Carneiro 80, Belo Horizonte, Minas Gerais 30510-010, Brazil; martadonascimento.phoneutria@gmail.com; 3 Faculty of Medicine, Minas Gerais Federal University, Neuroscience Laboratory, Av. Alfredo Balena 190, Belo Horizonte, Minas Gerais 30130-100, Brazil; romanosilva@gmail.com; 4 Department of Dentistry, Montes Claros State University, University Hospital, Health Laboratory Research, Montes Claros, Montes Claros, Minas Gerais 39401-001, Brazil; andreluizguimaraes@gmail.com

**Keywords:** Neuroprotection, retina, phoneutria nigriventer, spider toxins, PhTx3-4

## Abstract

The *in vivo* neuroprotective effect of PhTx3-4, a spider toxin N-P/Q calcium channel blocker, was studied in a rat model of NMDA-induced injury of the retina. NMDA (*N*-Methyl-d-Aspartate)-induced retinal injury in rats reduced the b-wave amplitude by 62% ± 3.6%, indicating the severity of the insult. PhTx3-4 treatment increased the amplitude of the b-wave, which was almost equivalent to the control retinas that were not submitted to injury. The PhTx3-4 functional protection of the retinas recorded on the ERG also was observed in the neuroprotection of retinal cells. NMDA-induced injury reduced live cells in the retina layers and the highest reduction, 84%, was in the ganglion cell layer. Notably, PhTx3-4 treatment caused a remarkable reduction of dead cells in the retina layers, and the highest neuroprotective effect was in the ganglion cells layer. NMDA-induced cytotoxicity of the retina increased the release of glutamate, reactive oxygen species (ROS) production and oxidative stress. PhTx3-4 treatment reduced glutamate release, ROS production and oxidative stress measured by malondialdehyde. Thus, we presented for the first time evidence of *in vivo* neuroprotection from NMDA-induced retinal injury by PhTx3-4 (-ctenitoxin-Pn3a), a spider toxin that blocks N-P/Q calcium channels.

## 1. Introduction

*N*-Methyl-d-Aspartate (NMDA) basically induces excitotoxicity contributing to damage in stroke and neurodegenerative retinopathies, leading to blindness, such as diabetic retinopathy, glaucoma, retinal vascular occlusion, optic nerve neuropathy, and retinopathy of prematurity [1,2].

The etiology and pathogenesis of these neurodegenerative diseases are different; however, at the cellular and molecular level, retinal injury is present in all of them [3]. The excitotoxicity injury consists of a self-reinforcing destructive cascade involving neuronal depolarization, calcium influx and oxidative stress that are initiated by energy failure and increased glutamatergic stimulation [4].

The electroretinogram (ERG) is a composite signal generated by the retina in response to light stimuli. The b-wave of the ERG is a particularly sensitive index of retinal injury of the retina, and ERG can be used to evaluate the efficacy of therapeutic drugs.

Glutamate is the major excitatory retinal neurotransmitter and is released *in vivo* by photoreceptors, bipolar cells and ganglion cells [5]. In the retinal injury, excessive amounts of glutamate are released and lead to neuronal cell toxicity [6]. Experimentally, in adult rats, intravitreal administration of glutamate causes retinal toxicity [7]. Evidences indicates that glutamate initiates neurotoxic cascades by several different mechanisms, most of which depend on [Ca^2+^]*_i_* increases. The activation of NMDA receptors depolarizes neurons and subsequently activates voltage-dependent Ca^2+^ channels (VDCCs) [8]. The net result of this process is an increase in the cytoplasmic calcium concentration, which activates proteases, nucleases, phospholipases, nitric oxide synthase and other degradative enzymes. This activation leads to an increase in free radicals production and subsequent cell death [9,10]. Several studies reported that ROS, including superoxide radicals, induces oxidative stress in many neurodegenerative diseases, including optic neuropathies.

An excessive influx of Ca^2+^ that causes an intracellular overload of Ca^2+^ in neurons is a crucial step for excitotoxicity, causing a large release of glutamate and enhanced production of ROS. Ca^2+^ overload, glutamate release and ROS formation are the major pathophysiological processes that contribute to retina injury. Therefore, one method to prevent the harmful effects of injury might be to block the excessive influx of calcium using calcium channel blockers.

PhTx3-4 is a toxin purified from the spider *Phoneutria nigriventer* venom that blocks N and P/Q voltage gated calcium channels with a similar potency [11,12]. R-type currents can also be inhibited by PhTx3-4, but with a lower potency [11]. Multiple binding sites appear to be involved in the PhTx3-4 action in the central nervous system. PhTx3-4 inhibits calcium influx [13,14] and exocytosis in synaptosomes by targeting P/Q calcium channels in nerve endings and glutamate release in synaptosomes [15]. Previously, we have shown that PhTx3-4 reduced the glutamate content and cell death of retinal ischemic slices submitted to oxygen-deprived low glucose medium [16]. The aim of the present work was to study the *in vivo* intravitreal injection of the purified toxin, PhTx3-4, in an *in vivo* model of retinal injury induced by NMDA injection.

## 2. Results

### 2.1. PhTx3-4 Reverses NMDA-Induced Retinal Injury Disfunctions of the Retina

**Figure 1:**
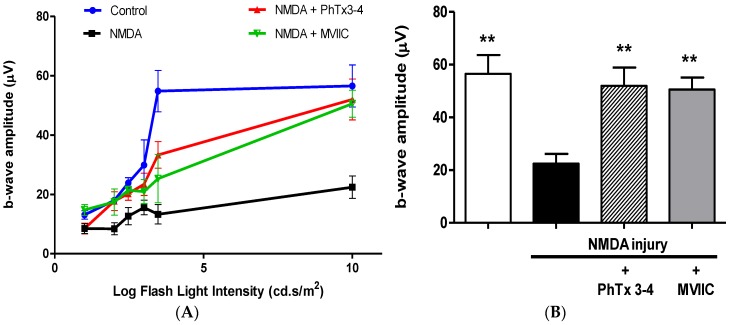
Functional evaluation of the photopic cone-driven electroretinogram responses to flashes. (**A**) The intensity-response function for the photopic b-waves amplitudes recorded in rats seven days after an intravitreal injection of saline (control), NMDA 200 nmol/eye injection (NMDA injury), co-administration of NMDA (200 nmol/eye) and PhTx3-4 (90 pmol/eye), co-administration of NMDA (200 nmol/eye) and MVIIC (30 pmol/eye). The points represent the means ± SEM; (**B**) Quantification of the amplitudes in 10 cd.s/m^2^ flash light intensity for the experimental groups described above. (Means ± SEM, **: *p* < 0.01, compared with the NMDA. A one-way ANOVA, followed by the Newman-Keuls post-test; *N* = 4–7 animals).

The retina b-wave amplitude is a very good indicator of the functional integrity of the retina [17]. The b-wave of the ERG is a particularly sensitive index of retinal injury of the ischemia [18]. The effect of the PhTx3-4 treatment, ω-conotoxin MVIIC treatment and no treatment of the retinas on NMDA-induced injury was initially evaluated by the amplitude of the retina b-wave of the ERG, Figure 1A. The quantification of the amplitude of the b-wave of ERG, using a flash light intensity stimulus of 10 cd.s/m^2^, is shown in Figure 1B. NMDA-induced injury of the retinas reduced the b-wave amplitude of the retinas by 62% ± 3.6%, whereas PhTx3-4 treatment and MVIIC treatment caused a reduction of only 9%± 2% and 12% ± 6%, respectively, which was no different from the control retinas that were not submitted to NMDA-induced injury, *p* > 0.05.

### 2.2. PhTx3-4 Causes Cell Protection of NMDA-Induced Injury of the Retina

**Figure 2:**
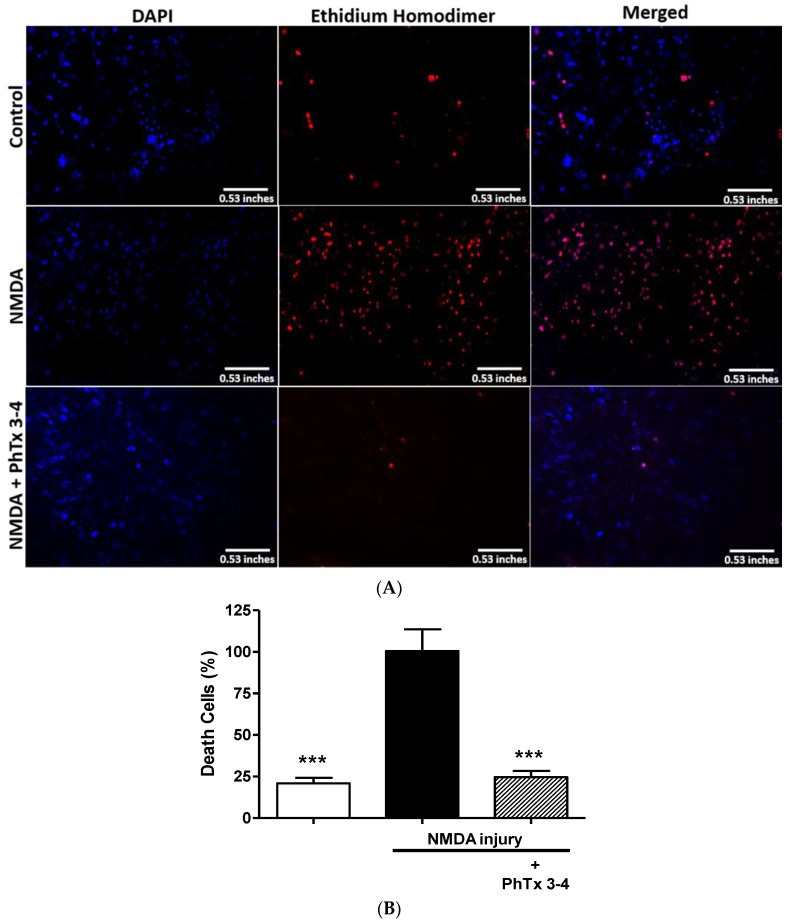
Neuroprotective effect of spider toxin PhTx3-4, seven days after NMDA injury. Following intravitreal injections of saline (control), NMDA 200 nmol/eye (NMDA injury) and NMDA-induced injury (200 nmol/eye) plus treatment with PhTx3-4, 90 pmol/eye, the retina was dissected and prepared for fluorescence microscopy images as indicated. (**A**) Representative retinal fluorescent images of the different experimental conditions (horizontal). In vertical, the applied protocol indicates cell nucleus in general (left panel, DAPI blue staining), nucleus of dead cells (middle panel, ethidium homodimer red staining) and merged images (right panel); (**B**) The graph shows the percentage of dead cells in retinal that were submitted to NMDA injury. One group was not treated with NMDA (open bars), group treated with NMDA (black bar), and group treated with 90 pmol/eye of PhTx3-4 and NMDA (hachured bar). Quantification of the results is expressed as the means ± SEM of dead cells in 10 fields of five different experiments. (***: *p* < 0.001, compared with the NMDA injury). A one-way ANOVA, followed by the Newman-Keuls post-test. *N* = 5 animals.

**Figure 3:**
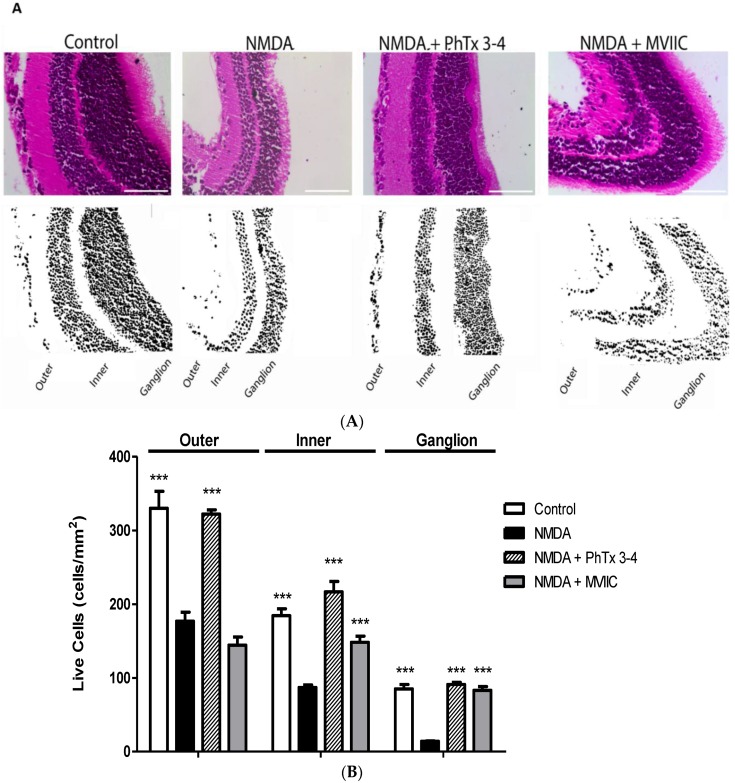
Neuroprotective effect of spider toxin PhTx3-4 in inner, outer, and ganglion cell layers of the retina. Control, NMDA-induced injury (200 nmol/eye), NMDA-induced injury (200 nmol/eye) plus treatment with PhTx3-4, 90 pmol/eye and NMDA-induced injury (200 nmol/eye) plus treatment with MVIIC, 30 pmol/eye are shown. (**A**) Representative micrographs of hematoxylin-eosin stained retina slices in each treatment group and binary mask after thresholding of each figure (scale bar 200 µm). Black pixels are the ROIs (regions of interest) and white pixels are background; (**B**) The graph shows the percentage of live cells in retina that were submitted to NMDA injury. Control group not treated with NMDA is shown in the open bars. Injury with NMDA (200 nmol/eye) is shown in the black bars. Treatment with 90 pmol/eye of PhTx3-4 or 30 pmol/eye of MVIIC is shown in hatched and grey bars, respectively. The results are expressed as the mean of the number of live cells on the respective layer from four different experiments. (mean ± SEM. ***: *p*<0.001, compared with the NMDA injury. A two-way ANOVA, followed by the Bonferroni post-test; *N* = 4 animals).

To evaluate whether PhTx3-4 functional protection of the retinas recorded on ERGs is accompanied by morphological protection of retina cells, fluorescence microscopy and cell imaging analysis of the retinas were performed. Figure 2 shows fluorescence microscopy images of the retinas submitted to a double staining for DAPI (4,6-diamidino-2-phenylindole), a fluorescent nuclear stain, and ethidium homodimer, a dead cells marker. Figure 2A shows representative images of control not subjected to injury, injury with NMDA and injury with NMDA treated with PhTx3-4. PhTx3-4 treatment of NMDA-induced injury of the retinas indicates robust protection against injury. The quantification of the number of dead cells in the NMDA-induced injury of the retinas treated/not treated with PhTx3-4 is shown in Figure 2B. The dead cells increased by 4.7-fold in the retinas as a result of NMDA-induced injury, while the PhTx3-4 treatment of the retina injury shows a reduced number of dead cells, which was not different from the control retinas without injury (2B), *p* > 0.05. To assess the injury damage to the cells of the retina layers, they were stained with hematoxylin-eosin, and the cells of the ganglion cell layer (GCL), inner cell layer (ICL) and outer cell layer (OCL) were counted. The thickness of the GCL, ICL and OCL were reduced by NMDA-induced injury of the retinas in comparison with the control group that was not submitted to injury. The live cells in the OCL, ICL and GCL in the control retinas without injury were 330 ± 23, 176 ± 7 and 85 ± 6, respectively. The NMDA-induced injury of the retinas reduced the live cells in these layers to 177 ± 12, 87 ± 3 and 14 ± 0.6 with a reduction of 46%, 51% and 84%, respectively, *p* < 0.001, Figure 3. The major reduction was seen in the GCL, and it was noteworthy that PhTx3-4 treatment of retinal injury shows no difference in the number of live cells in the retina layers to that not submitted to injury, control *p* > 0.05.

### 2.3. PhTx3-4 Treatment Reduces Glutamate Release in NMDA-Induced Injury of the Retinas

**Figure 4:**
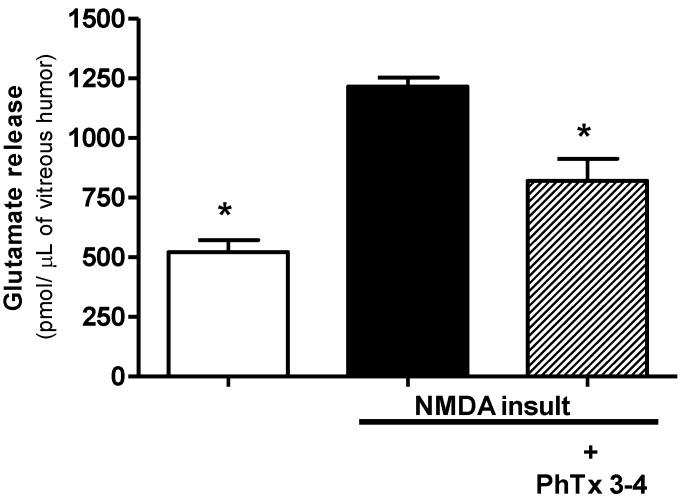
The effect of the spider toxin PhTx3-4 treatment on the glutamate release of the aqueous humor one day after the NMDA-induced injury of the retina. The glutamate release from the retinal cells collected in the aqueous humor of the control, NMDA-induced injury (200 nmol/eye) and NMDA-induced injury (200 nmol/eye) plus treatment with PhTx3-4, 90 pmol/eye is shown. The results are expressed as the means ± SEM of glutamate pmol/µL of the aqueous humor of five different experiments. (*: *p* < 0.05, a one-way ANOVA, followed by the Newman-Keuls post-test; *N* = 5–8 animals).

Glutamate is the neurotransmitter used by the synapses in the retina, and the release of this excitatory amino acid into the extracellular space increases with the retinal damage. NMDA-induced injury of the retina increased the glutamate release by 2.4-fold in the aqueous humor of the retina (Figure 4). The glutamate control value of the aqueous humor of the retinas without injury was 507 ± 47 pmol/µL and increased to 1218 ± 38 pmol/µL in the aqueous humor of the NMDA retinal injury. The treatment of retinal injury with 90 pmol PhTx3-4 reduced the glutamate release in the aqueous humor to 820 ± 92 pmol/µL, *p* < 0.01.

### 2.4. PhTx3.4 Treatment Reduces ROS in NMDA-Induced Injury of the Retinas

**Figure 5:**
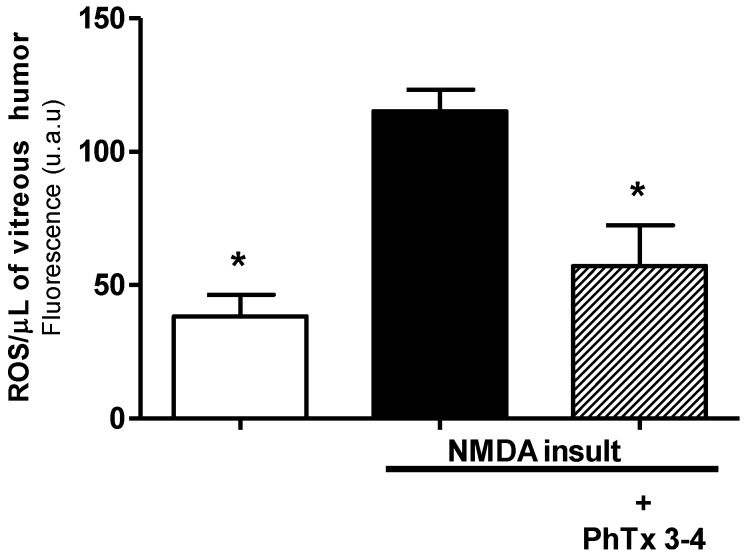
The effect of the spider toxin PhTx3-4 treatment on the ROS content of the vitreous humor one day after the NMDA injection. The ROS release from the retinal cells in the vitreous humor of the control, NMDA-induced injury (200 nmol/eye) and NMDA-induced injury plus treatment with PhTx3-4, 90 pmol/eye is shown. The results are expressed as the mean ± SEM of ROS/µL. (*: *p* < 0.05, compared with the NMDA injury, a one-way ANOVA, followed by the Newman-Keuls post-test; *N* = 6–7 animals).

An enhanced production of ROS is suggested to be one of the major pathophysiological processes that contribute to retinal damage. ROS production induced by NMDA-induced injury of the retina increased by 4.0-fold in the aqueous humor of the retina, Figure 5. The ROS control value for the retinas without injury was 28 ± 5 u.a/µL (arbitrary unit/ µL) and increased to 115 ± 8 u.a/µL in the NMDA-induced injury condition (*p* < 0.01). The PhTx3-4 treatment reduced the ROS production to 56 ± 14 u.a/µL, a value not different from the control retinas without injury (*p* > 0.05).

### 2.5. PhTx3-4 Treatment Reduces Oxidative Stress in NMDA-Induced Injury of the Retinas

**Figure 6:**
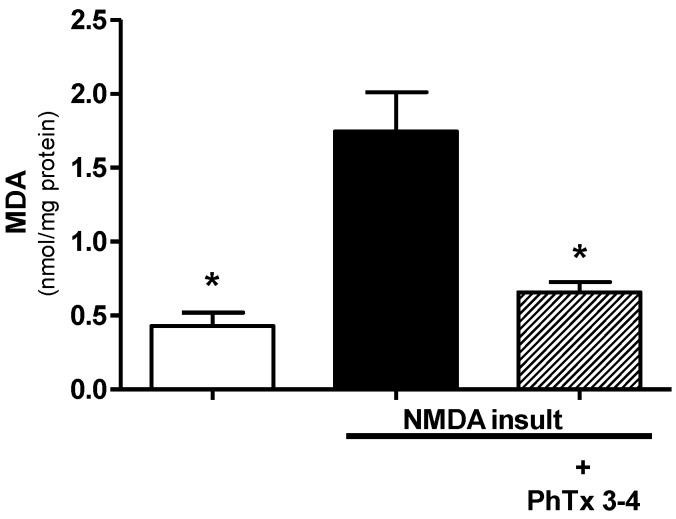
The effect of spider toxin PhTx3-4 treatment of the NMDA-induced injury of the retina on the MDA levels of the retina. MDA in the retina homogenates of the control, NMDA-induced injury (200 nmol/eye) and NMDA-induced injury (200 nmol/eye) plus treatment with PhTx3-4, 90 pmol/eye is shown. The results are expressed as the mean ± SEM of MDA (nmol/mg protein of retina). (* *p* < 0.05 compared with the NMDA injury. A one-way ANOVA, followed by the Newman-Keuls post-test; *N* = 5–7 animals).

The MDA levels in the retinal homogenates were used as a marker of oxidative stress of the retinas. Measurement of MDA levels is frequently used for determining the lipid peroxidation levels. MDA is not a specific or quantitative indicator of fatty acid oxidation but correlates with the lipid peroxidation level. NMDA-induced injury of the rat retinas increased MDA by 3.5-fold in the retina homogenates, Figure 6. Measurements were performed seven days after NMDA injection. The MDA control value of the retinas without injury was 0.49 ± 0.1 nmol/mg protein and increased to 1.7 ± 0.2 nmol/mg protein as a result of NMDA-induced injury. The PhTx3-4 treatment of the NMDA-induced injury of the retinas reduced the MDA to 0.65 ± 0.07 nmol/mg protein, which was not different from the retinas’ homogenates without NMDA-induced injury, the control, *p* > 0.05. Thus, PhTx3-4 treatment reduced oxidative stress of the retinas submitted to NMDA injury.

## 3. Discussion

Many retinal disorders such as retinal ischemia-reperfusion, glaucomatous, diabetic retinopathy and traumatic optic neuropathy are associated with excitotoxic neuronal cell death [19,20]. Glaucoma is an ischemic optic neuropathy with functional changes and structural features in the visual field in the optic nerve head [21]. It is considered the second most common cause of preventable blindness worldwide [22]. Diabetic retinopathy (DR) is one of the most common complications of diabetes mellitus and is the second leading cause of blindness in the world. [23,24]. The reduction of blood flow in the retina has been described as the first modification that occurs in these patients. As the vascular lesion progresses, a wide ischemic area develops, at which neovascularization of the retina and optic nerve may become evident, as reviewed by Giuliari in 2012 [25]. Retinal ganglion cell (RGC) death is a common feature of many ophthalmic disorders (such as glaucoma, and central retinal artery or vein occlusion) and may occur via a variety of mechanisms involving, for example, excitatory amino acids [26], nitric oxide [27], apoptosis [28] and reduced retinal perfusion. In most of the situations above, a large release of glutamate together with an intracellular calcium overload and enhanced production of free radicals are suggested to be three major pathophysiological processes that contribute to retinal damage.

The electroretinogram (ERG) is a complex change in potential, in response to a light stimulus, which is recorded across the eye, or more directly across the retina. The b-wave amplitude of the ERG is a particularly sensitive index of retinal damage [29]. The b-wave is the ERG-component most susceptible to ischemia [30]. This electrical wave is induced by potassium efflux shunted from “on” bipolar cells in the vitreous humor and by the Müller cells in response to retinal illumination. Experimental conditions using different models of retinal damage show a reduction of the b-wave amplitude. Thereby, the functional status of the retina can be monitored continuously by recording the ERG [29]. The b-wave of the ERG represents a functional measure for the potential therapeutic efficacy of drugs interacting with the pathophysiological processes of the retina. In the present study, we induced retinal injury of the rats’ retinas by intravitreal injection of NMDA and tested the therapeutic action of PhTx3-4 and MVIIC, both N-P/Q calcium channel blockers, on the pathophysiological processes found in retina injury. The PhTx3-4 treatment of NMDA-induced injury of the retinas significantly recovered the b-wave amplitude of the retina, which decreased as a result of NMDA-induced injury. The treatment with ω-conotoxin MVIIC, another high voltage activated calcium channel blocker, showed a similar response in the recovery of b-wave amplitude.

Glutamate is the major neurotransmitter of the retina where ischemic and excitotoxic lesions were described in several retinal pathologies, such as diabetic retinopathies and glaucoma. To ensure reliable signal transmission, the synaptic glutamate concentration in the retina must be regulated by rapid removal of free neurotransmitters from the cleft by glutamate transporters [31]. Glutamate may be considered a potent neurotoxin [32] and is responsible for degenerative injuries of the central nervous system [33,34].

By the glutamate-Ca^2+^ neurotoxicity hypothesis [35], the excessive release of glutamate causes a pathologic elevation in the intracellular Ca^2+^ concentration, which activates a calcium-dependent process that leads to cell death. PhTx3-4 treatment of NMDA-induced injury of the retinas reduced glutamate release and excitotoxicity, demonstrating strong protection of the cells with a virtual exclusion of the dead cells observed in the injury condition. This result indicates that PhTx3-4, an N-P/Q Ca^2+^ channel blocker, protects neuronal cells against retinal neurotoxicity both *in vivo* and *in vitro* [16], and may be useful as a therapeutic drug against retinal diseases that cause neuronal injury.

Glutamate receptor activation induces excitotoxicity and has been hypothesized to cause retinal ganglion cell death in glaucoma and to link mitochondrial dysfunction in both acute and chronic neurodegenerative disorders [33]. Another factor that contributes to the maintenance of excitotoxicity is the glutamate overspill throughout the reversal of the glutamate transporters during ischemia [36]. PhTx3-4 treatment of the NMDA-induced injury had a remarkable neuroprotective effect on the cells of the retina layers, and the major neuroprotection action was on the ganglion cell layer (GCL), which was more susceptible to injury damage. MVIIC treatment of the NMDA-induced injury did not achieve the same efficiency of the spider toxin; it is not able to reverse the loss of retinal outer layer and is less effective in reducing cell death in the inner cell layer. A mortality of 84% in the GCL layer was found from the NMDA-induced injury. A high mortality of GCL of the retina has been described, using a variety of methods to induce retinal injury damage. In an *in vivo* rat model of is chemic damage of the retina, induced by elevating intraocular pressure, a 70% reduction in GCL was described [37]. Blocking the Ca^2+^ channel provided protection for the retinal GCL from NMDA-induced excitotoxicity [8]. It was described that N-type Ca^2+^ channels are expressed in the OCL, ICL and GCL of the retina [38]. N-type VDCCs represent 40% of the whole-cell Ca^2+^ currents in adult rat GCL [39], which express functional NMDA receptors [40]. Retinal ganglion cells have voltage-gated ion channels that are permeable to Ca^2+^ ions [41], including Q-type calcium channels [42]. The neuroprotective effect of PhTx3-4 on NMDA-induced injury of the retina might be due to its action of blocking N-P/Q calcium channels [11] and its action of blocking glutamate transporters, a target that has been previously associated with PhTx3-4’s mechanism of action [43,44]. This could explain the differential neuroprotective action of PhTx3-4 and MVIIC in our model, since MVIIC does not act in glutamate transporters. L-type calcium channel blockers were suggested to be *in vivo* neuroprotective agents after ischemic injuries of the retina [45] however, we cannot exclude the contribution of the regulation of blood flow to explain the neuroprotection induced by L-type calcium channels.

Many studies have provided evidence that ROS are important contributors to neuronal injury mediated by ischemia. The pathogenesis of retinal ischemia has been associated with a depletion of cellular energy sources, a massive release of excitatory amino acids, mitochondrial dysfunction and the formation of reactive oxygen species that contribute to oxidative damage [46,47]. Mitochondrial dysfunction, superoxide generation and oxidative stress play a significant role in the mechanisms underlying retinal degeneration in ocular disease states, such as glaucoma, age-related macular and ischemia. The retina is considered to be an ideal model for examining ROS-mediated pathological events, due to the high content of polyunsaturated fatty acids and high oxygen consumption [47,48]. Free radicals formed during oxidative stress can directly attack polyunsaturated fatty acids and initiate free radical chain reactions that result in lipid peroxidation in cellular membranes.

In this *in vivo* study, we demonstrated that PhTx3-4, an N-P/Q calcium channel blocker, significantly recovered the b-wave amplitude of the retina that decreased as a result of NMDA-induced injury. PhTx3-4 also was capable of reducing glutamate excitotoxicity, oxidative stress, retinal cell death and free radical production, pathophysiological processes involved in retinal injury of the retina. The results hold significant promise for PhTx3-4’s role as a novel therapeutic agent to manage symptoms of retinal injury of the retina.

## 4. Materials and Methods

Male Wistar rats (180–220g) were used in this study. Five rats were used in each experiment. All of the experiments were performed in accordance with the current guidelines for the care of laboratory animals. The Ethics Committee of the Federal University of Minas Gerais, CEUA, authorized the studies (Protocol number 347/2012), in 14 March 2013. We followed the guidelines for the Use and Care of Animals for Research issued by the NIH.

### 4.1. Drugs

PhTx3-4 was purified using a combination of gel filtration, reverse-phase FPLC (GE, MN, USA) ion exchange HPLC(Shimadzu, Tokyo, Japan), as described previously. Pro-Rpc columns, reverse phase high-performance liquid chromatography (HPLC) on Vydac C18, and ion exchange HPLC on cationic and anionic columns [49,50]. By mass spectroscopy analysis, PhTx3-4 has a molecular weight of 8449 DA, and the amino acid sequence is: SCINVGDFCDGKKDCCQCDRDNAFCSCSVIFGYKTNCRCEVGTTATSYGICNAKHKCGRQTTCTKPCLSKRCRRNHG, accession NCBI P81790. The predicted mass of the sequence of PhTx3-4 is 8419 and the mass spectroscopy analysis is 8449 DA. The difference may be due to the fact that when a proline residue of peptide is not stabilized by disulfide bridges, artificial components are generated by the mass spectrometer conditions, due to the cleavage of the peptide bond at the proline position [51]. The snail toxin ω-conotoxin MVIIC was obtained from Peptides, Osaka, Japan. All other chemicals were of analytical grade.

### 4.2. Retinal Injury

Retinal injury of the retinas was induced by intravitreal injection of 200 nmol of NMDA in a volume of 5 µL [52]. The injection was performed with a 10 µL Hamilton micro syringe inserted 1 mm below the corneal limbus through the infero-temporal sclera. Rats were under anesthesia in a chamber with 4% halothane in 25% oxygen.

Two hundred nanomolar of NMDA were injected into the vitreous cavity of the right (injury group). Contralateral eye was treated with co-administration of PhTx3-4 (90 pmol/eye) and NMDA (200 nM) or with MVIIC (30 pmol/eye) + NMDA (200 nM). In other rats, 5 µL of saline was injected in one eye (control), and 200 nmol NMDA was injected in the contralateral eye (injury group). At 7 days after the intravitreal injection of NMDA, the eyes were evaluated.

### 4.3. Electroretinography

After 7 days of the NMDA-induced injury, electroretinogram (ERG) recordings of the retina in control (saline treatment), injury, injury/ PhTx3-4 and injury/MVIIC treated were performed. General anesthesia was induced by an intraperitoneal injection of a mixture of ketamine (50 mg/kg) and xylazine (8 mg/kg). The pupils were dilated with a mixture of tropic amide (Mydriaticum; Alcon, São Paulo, Brazil) and phenylephrine (Allergan, São Paulo, Brazil). The anesthetized rats were placed in a ganzfeld bowl on a sliding table (Electrophysiological diagnostic systems-Professional Series P227f, Roland Consult, Brandenburg, Germany). The body temperature was maintained at 38 °C by means of a heating pad and monitored rectally. Silver needle electrodes served as the reference (forehead) and ground (tail), while gold wire rings were used as the active electrodes. An ophthalmic 2% methylcellulose solution (Mediphacos, Belo Horizonte, Brazil) was applied to the cornea to keep it hydrated for the electrical conductance. In previous photopic ERG sessions, the rats were subjected to adapt to the light background of 25 cd.s/m^2^. cd.s/m^2^ (candela.seconds/squared methods) is the unit for luminous intensity of the international system of units. The intensity of the light responses was 0.01, 0.1, 0.3, 1, 3, and 10 cd.s/m^2^. The ERG was used to measure the amplitude of the b-wave, and the results were presented as the mean compared with the average values of all of the animals [53].

### 4.4. Fluorescence Microscopy and Imaging Analysis

To analyze the cell viability, the rats were killed by decapitation, and the retinas were removed and double stained with 6 µmol/L ethidium homodimer-AM and 300 nM DAPI for 30 min. Then, they were washed for 15 min in 2 mL of PBS (Phosphate Buffer Saline), 95% O_2_/5% CO_2_ at room temperature. Cells nuclei showed blue fluorescence of DAPI (4,6-diamidino-2-phenylindole) and dead cells were indicated by the red fluorescence of ethidium homodimer. The images were acquired using an Axiovert Zeiss 200 M (Zeiss, Oberkochen, Germany) using the ApoTome (Zeiss, Oberkochen, Germany). The objectives were used without immersion at a 20× magnification. Imaging analysis was performed as previously described [54]. The images showing specific red-fluorescent nucleic acid staining of the dead cells were used to quantify the NMDA-induced neurotoxicity. We used the Image J software (1.6.0, National Institute of Mental Health, Maryland, USA, 2015) to combine consecutive optical sections from a Z-series to create image reconstructions. To improve the images for the quantitative analysis, they were processed using the median filter. In the current approach, we define the nucleus as connected pixels that were above a threshold. This threshold was calculated using the image histogram, and pixels below the threshold were set to 0.

### 4.5. Histopathology Study

The retinas were fixed in 4% formaldehyde in 10 mM of phosphate-buffered saline and rinsed with phosphate-buffered saline. Then, they were stained with hematoxylin-eosin to assess the NMDA damage on the cells of the ganglion cell layer (GCL), inner nuclear layer (ICL), and outer nuclear layer (OCL). Conventional microscopy using Olympus^®^ BH2 microscope (Olympus, Tokyo, Japan), 40× objective and software Image J. The live cells were analyzed in 20 fields per retinal slice. The results were expressed as the mean of the live cells per square millimeter.

### 4.6. Glutamate Assay

The glutamate content of the vitreous humor of the control, NMDA injury and NMDA injury PhTx3-4 treated eyes were determined by monitoring the increase in the NADPH+ (1.0 mM) fluorescence in the presence of glutamate dehydrogenase (50 units), as previously described [55]. The excitation wavelength was set to 340–360 nm, and the emission wavelength was set to 450 nm in a Shimadzu spectrofluorometer (Kyoto, Japan).

### 4.7. Free Radicals (ROS) Content of the Vitreous Humor

The ROS measurements of the vitreous humor were performed by using DCF-DA, 2′,7′-dichlorofluorescein diacetate (Sigma-Aldrich (St. Louis, MO, USA) a fluorescent probe for the assay [56]. Briefly, 2 µL of the vitreous humor was incubated with 100 µL of 125-µM DCFH-DA stock solution at 37 °C for 30 min and protected from light. The formation of the oxidized fluorescent derivative (DCF) was monitored at excitation and emission wavelengths of 488 and 525 nm, respectively, in a fluorescent plate reader (PerkinElmer, Waltham, MA, USA).

### 4.8. Lipid Peroxidation

Lipid peroxidation was determined by measuring the malondialdehyde (MDA) content in the retina homogenates [57]. Malondialdehyde is the principal and most studied product of polyunsaturated fatty acid peroxidation. Briefly, thiobarbituric acidic reacts with MDA at high temperatures (95–100 °C), and this reaction product was measured at 532 nm using a spectrophotometer (Ultrospec 2100, Amersham, Piscataway, NJ, USA). The results were normalized by the protein content and expressed as the percentage of increase of MDA relative to their respective controls. Measurements of MDA were performed 7 days after NMDA injection.

### 4.9. Data Analysis

The experiments of cell viability and biochemical tests were analyzed with a one-way ANOVA followed by the Newman Keuls test. The results were expressed as the mean ± SEM from at least five independent experiments. *p* < 0.05 was considered statistically significant.
